# Normal saline as resuscitation fluid in critically ill patients: not dead yet!

**DOI:** 10.1186/s13613-016-0227-4

**Published:** 2016-12-28

**Authors:** Patrick M. Honore, Rita Jacobs, Herbert D. Spapen

**Affiliations:** ICU Department, Universitair Ziekenhuis Brussel, Vrije Universiteit Brussel, 101, Laarbeeklaan, 1090 Brussels, Belgium

In this issue of Annals of Intensive Care, Van Regenmortel et al. report a significant association between hyperchloremia present on the first day of intensive care unit (ICU) admission and ICU and hospital mortality in a large cohort of ICU patients. However, chloride levels were significantly higher and mortality was lower in cardiac surgery patients than in the entire cohort [1].

Hyperchloremia is increasingly stigmatized to negatively influence kidney function and mortality in ICU patients. Paradoxically, normal saline (NaCl 0.9%; NS)—the worldwide number one solution for patient resuscitation purposes—contains supraphysiologic concentrations of chloride and, when amply infused, inevitably causes hyperchloremic metabolic acidosis (HMA) [2]. Animal models typically highlight the adverse and unwarranted effects of NS-induced HMA such as greater hemodynamic instability, altered microcirculation, and enhanced or persistent inflammation [3, 4]. Growing aversion for NS produced a surge of studies comparing NS with different “balanced” crystalloid solutions. The latter (i.e., Ringer’s solution, Hartmann’s solution, and Plasma-Lyte^®^) more closely resemble the electrolyte component of plasma but, most importantly, contain much less chloride than NS (109, 111, and 98 mmol/L, respectively).

Basically, the case against NS is entirely built on its disruptive effect on acid–base homeostasis. Among the different viewpoints on acid–base equilibrium, the Stewart–Figge approach explains NS-induced acidosis by an increase in chloride anion concentration. According to this method, the apparent strong ion difference (SIDa) is an independent variable that is unaltered by unidirectional sodium and chloride changes and thus a more appropriate detector of fluid-related HMA [5]. As the SIDa of NS equals zero, infusion of NS will decrease plasma SIDa and contribute to metabolic acidosis. The observation by Van Regenmortel et al. that a decreased SIDa (excluding lactate) did not have an impact on mortality thus argues against a deleterious infusion-related role of chloride *in se* [1].

Several arguments strongly plead against a too precipitous verdict on NS. A randomized, controlled, double-blind crossover study comparing the effects of a 2 L infusion of 0.9% NS versus Plasma-Lyte^®^ in healthy volunteers demonstrated significant reductions in mean renal artery flow velocity (*P* = 0.045) and renal cortical tissue perfusion (*P* = 0.008) from baseline after NS. However, there was no evidence of relevant acute kidney injury (AKI) as shown by similar concentrations of urinary neutrophil gelatinase-associated lipocalin in both infusion groups [6]. A meta-analysis assessing high- versus low-chloride infusion in 6253 peri-operative and critically ill patients found a significant but weak association between high-chloride content fluids and a higher incidence of AKI. Excluding the most heavy-weighted studies from this analysis, however, rendered the AKI end point non-statistically significant [7]. The subsequently published 0.9% Saline versus Plasma-Lyte^®^ for Intensive Care Unit Fluid Therapy trial found no difference in incidence of AKI, renal replacement therapy and mortality among patients receiving similar volumes of NS or buffered crystalloid [8]. Moreover, a very recent cluster-randomized crossover study comparing the administration of similar volumes of NS and balanced crystalloids in nearly 1000 patients showed no difference in a composite “major kidney-related adverse events” end point including death, dialysis, and persistent renal dysfunction [9]. Finally, theoretically beneficial effects of NS-induced acidosis such as enhanced tissue oxygen extraction and better protection against hypoxemia have insufficiently been investigated [10].

An inherent but important limitation clouding the retrospective data of Van Regenmortel et al. is the inability to correctly determine the fluid type and volume before ICU admission. Indeed, irrespective of the type of admission (medical, surgical, or trauma) and after adjustment for important confounders (illness severity and comorbidities), the need to acutely administer a high volume of fluid or a positive fluid balance over time is independently and reproducibly associated with more ICU complications (including AKI [11, 12]) and higher mortality [13], especially in patients with underlying kidney or heart disease [14]. In addition, many questions remain unsolved. Are some patient subgroups more chloride-vulnerable? Is a “dose” limitation of NS (e.g., not exceeding 20–30 mL/kg) advised? Are pre-resuscitation chloride concentrations important? Does hyperchloremia only matter when associated with SIDa changes or a particular degree of acidemia? Are all balanced crystalloids equivalent with regard to relevant clinical outcome parameters?

The study of Van Regenmortel et al. indirectly underscores growing equipoise in the expert medical community regarding the presumed harmful effects of NS compared with balanced crystalloids. Future studies, such as the ongoing multicenter randomized Plasma-Lyte^®^ versus Saline (PLUS) trial, should hopefully definitively unravel the crystalloid conundrum ate.

## Abbreviations

ICU: intensive care unit
NS: normal saline
HMA: hyperchloremic metabolic acidosis
SIDa: apparent strong ion difference
AKI: acute kidney injury
